# Comparative Transcriptomic Profiling of Mesenchymal Stem Cells from Distinct Tissue Origins and Isolation Methods Highlights the Stability and Immunomodulatory Signature of Umbilical Cord-Derived Smumf Cells

**DOI:** 10.1007/s13770-025-00765-2

**Published:** 2025-11-24

**Authors:** Min Ji Lee, Kyungtaek Park, Sungho Won, Chris Hyunchul Jo

**Affiliations:** 1 Department of Orthopedic Surgery, SMG-SNU Boramae Medical Center, Seoul National University College of Medicine, 20 Boramae-ro 5-gil, Dongjak-gu, Seoul, 07061 Korea; 2 Department of Translational Medicine, Seoul National University College of Medicine, 103 Daehak-ro, Jongno-gu, Seoul, 03080 Korea; 3 Institute of Health and Environment, Seoul National University, 1Gwanak-ro, Gwanak-gu, Seoul, 08826 Korea; 4 Interdisciplinary Program of Bioinformatics, College of Natural Sciences, Seoul National University, 1Gwanak-ro, Gwanak-gu, Seoul, 08826 Korea; 5 Department of Public Health Sciences, Graduate School of Public Health, Seoul National University, 1Gwanak-ro, Gwanak-gu, Seoul, 08826 Korea; 6 Institute of Reproductive Medicine and Population, Medical Research Center, Seoul National University, 103 Daehak-ro, Jongno-gu, Seoul, 03080 Korea; 7 RexSoft Corp, 1Gwanak-ro, Gwanak-gu, Seoul, 08826 Korea; 8 Department of Statistics (Institute of Applied Statistics), Jeonbuk National University, 567 Baekje-daero, Deokjin-gu, Jeonju, 54896 Korea

**Keywords:** Mesenchymal stem cells, RNA sequencing, Immunomodulation, Cell therapy

## Abstract

**Background:**

Mesenchymal stem cells (MSCs) derived from bone marrow (BM), adipose tissue (AD), and umbilical cord (UC) exhibit therapeutic potential in regenerative medicine. However, their properties, including transcriptomic profiles, vary based on tissue origin, passage stage, and isolation method, complicating their clinical standardization. Addressing these unresolved differences requires comprehensive approaches, such as RNA sequencing, to analyze transcriptomic profiles in detail.

**Methods:**

In this study, RNA-seq was employed to analyze MSC transcriptomes from BM, AD, and UC tissues. UC MSCs were isolated using enzymatic digestion or the Minimal Cube Explant (MCE) method (smumf cells), and transcriptomes of early (P3–4) and late (P10) passages of smumf cells were compared. Differentially expressed genes (DEGs) were identified, followed by transcription factor (TF) and pathway analyses.

**Results:**

Fetal MSCs (UC and smumf cells) exhibited distinct transcriptomic profiles compared to adult MSCs (BM and AD), with 2,208 upregulated and 2,594 downregulated DEGs. Key transcription factors, such as E2F1 and NF-κB1, and pathways, including glycolysis, cholesterol biosynthesis, and TNF-α signaling, were enriched in fetal MSCs. smumf cells demonstrated transcriptomic stability between early and late passages, with only 12 DEGs identified. Additionally, smumf cells showed enhanced innate immune responses and cholesterol metabolism compared to enzymatically isolated UC MSCs.

**Conclusion:**

This study provides a comprehensive transcriptomic comparison of MSCs, highlighting the superior transcriptional stability, immunomodulatory capacity, and metabolic flexibility of fetal MSCs, particularly smumf cells. These findings underscore their potential as a reliable cell source for therapeutic applications and encourage further exploration of their clinical application.

**Supplementary Information:**

The online version contains supplementary material available at 10.1007/s13770-025-00765-2.

## Introduction

Mesenchymal stem cells (MSCs) have therapeutic potential due to their ability to differentiate, promote tissue repair, modulate immunity, and reduce inflammation in musculoskeletal and immune-related disorders [1–5]. However, their characteristics vary depending on the tissue of origin—adipose tissue (AD), umbilical cord (UC), or bone marrow (BM). MSCs can be derived from fetal tissues (e.g., umbilical cord, umbilical cord blood, placenta), or adult tissues (e.g., bone marrow, adipose tissue). Fetal MSCs exhibit superior proliferation, differentiation potential, and immunomodulation compared to adult MSCs, and they are less affected by age-related declines [6–9]. UC MSCs, in particular, are non-invasive to collect and can be expanded efficiently, unlike BM MSCs, which are limited by their invasive collection method and age-related reduction in yield [10–14]. While BM MSCs remain the most studied, alternative sources like AD MSCs are easier to obtain and offer comparable properties [15–17]. Despite the advantages of fetal MSCs, concerns about their stability, quality, and long-term functionality in clinical applications require thorough characterization. Therefore, the choice of MSC source should be carefully considered based on the specific therapeutic application.

Establishing protocols to isolate high-quality MSCs while minimizing senescence is essential for clinical applications [18]. Isolation methods and the passages at which they are utilized further influence their characteristics and potential clinical applications [2, 18]. Prolonged culture and replicative exhaustion can compromise MSC characteristics, leading to inconsistent clinical outcomes [19]. Expanding MSC cultures while preserving their quality is crucial, as achieving a clinical dose requires a large number of cells. UC MSCs are particularly noted for their ability to maintain stem cell properties during extended *in vitro* culture. However, transcriptional changes have been observed during prolonged culture, with significant alterations occurring after passage 5 and accelerating beyond passage 9 [19].

To address these challenges, UC MSCs can be isolated using enzymatic digestion or the explant method. The explant method avoids enzyme-induced cell damage and reduces biomaterial processing time [13]. In our previous study, we optimized the explant method, referred to as the ‘Minimal Cube Explant’ (MCE) method, and determined that 2–4 mm UC tissue pieces provide optimal conditions for MSC isolation. The MSCs isolated using this protocol were termed ‘smumf cells’ (small umbilical cord-derived fast proliferating cells), reflecting their origin and rapid proliferation capacity. This approach yielded a higher initial MSC count and greater bioactive factor abundance compared to enzymatic digestion [18]. MSCs isolated via the MCE method, termed smumf cells, exhibited robust proliferative capacity, retaining genomic stability from P3 to P10 and showing no significant increase in senescence markers, such as p16, p21, and p53, until passage 10. SA-β-gal activity also remained stable until passage 14 [20]. Maintaining cell integrity, including genomic stability and stemness, is vital to ensuring MSC stability and therapeutic suitability during long-term culture. Characterizing early- and late-passage MSCs, along with evaluating how isolation methods influence their properties, is essential for clinical applications. However, differences in mRNA expression profiles between smumf cells and enzymatically isolated UC MSCs, as well as between early and late passages, remain uncharacterized.

Addressing these unresolved differences in MSC properties requires advanced approaches, such as RNA sequencing, to provide a comprehensive understanding of their transcriptomic profiles. RNA sequencing (RNA-seq) on next-generation sequencing (NGS) platforms has significantly advanced whole transcriptome analysis, enabling the complete annotation and quantification of numerous genes in a single run. In this study, we utilized RNA-seq to analyze whole transcriptomes across 3 key aspects: (1) tissue sources, including BM MSCs, AD MSCs, and UC MSCs; (2) UC MSCs isolated via enzymatic digestion versus the Minimal Cube Explant (MCE) method; and (3) early and late passages of UC MSCs. This comprehensive comparative analysis aims to provide deeper insights into the impact of tissue origin, passage stages, and isolation techniques on MSC characteristics.

## Materials and methods

### smumf cells and UC MSC isolation and culture

This study was approved by the Institutional Review Board at Seoul Metropolitan Government Seoul National University Boramae Medical Center (IRB No.06–2012–78). Informed consent was obtained from all patients before performing the study. Human umbilical cords were obtained from full-term birth via cesarean section. The umbilical cords were washed 3 times with Dulbecco’s phosphate-buffered saline (DPBS; Welgene, Daegu, Korea) to remove blood products, and the length and weight were measured. UC MSCs were isolated through 1) MCE method, smumf cells; 2) enzymatic method, UC MSCs.

smumf cells were isolated according to the previous report, using the Minimal Cube Explants (MCE) method, in which umbilical cord tissue was cut into 2–4 mm cubes using surgical scissors [18]. In brief, smumf cells were isolated by cutting the entire umbilical cord tissue into 2–4 mm pieces (designated as MCE 2–4) and placed in 150 mm culture dishes (1 g/dish). After allowing firm tissue attachment for 1 h in a 37 °C, 5% CO_2_ incubator, culture medium was gently added. The medium used was low-glucose Dulbecco’s modified Eagle’s medium (LGDMEM; Hyclone, Logan, USA) supplemented with 10% fetal bovine serum (FBS; Hyclone), 100 U/mL penicillin, 100 μg/mL streptomycin, and 0.25 μg/mL amphotericin B (Welgene). Once cells reached 80% confluency, they were detached using a 3-min incubation with trypsin–EDTA, washed, and replated at the same density. The medium was replaced twice weekly, removing non-adherent cells in the process. Upon reaching 80% confluency, cells were detached using 0.05% trypsin and 0.53 mM EDTA (Welgene) for 3 min. The tissues were filtered through a 100-μm cell strainer (SPL Life Sciences, Pocheon, Korea), and the cells were centrifuged at 500 g for 5 min at 20 °C before being replated at a density of 3 × 10^3^ cells/cm^2^. Cells were cryopreserved at passage 3 or 8, and prior to experiments, thawed cells were expanded once and used at the subsequent passage. Before use in experiments, the smum cells were characterized by morphology, growth kinetics, CFU-F assay, flow cytometry, and trilineage differentiation, as previously reported [18]. Early passages from three to four and late passage of ten were used to further analysis.

UC MSCs were enzymatically isolated by treating with 0.1% collagenase type I (C0130, Sigma Aldrich, St. Louis, MO, USA) in low-glucose Dulbecco’s modified Eagle’s medium (LGDMEM; Hyclone, Logan, USA) supplemented with 10% fetal bovine serum (FBS; Hyclone) and 100 U/mL penicillin, 100 μg/mL streptomycin, and 0.25 μg/mL amphotericin B (Welgene) for 2 h at 37 °C with 60–70 rpm. Undigested tissues were removed using a 100-μm cell strainer (SPL Life Sciences, Pocheon, Korea), and isolated cells were washed twice. Cells were resuspended in LG-DMEM supplemented with 10% FBS and antibiotic–antimycotic solution, and plated at a density of 1 × 10^4^ cells/cm^2^ in a 5% CO_2_ incubator at 37 °C. The culture medium was changed every 2–3 days and continuously cultivated. When cells reached 80% confluency, cells were detached by trypsin–EDTA for subculture. UC MSCs at passage four to five were used.

### BM MSCs isolation and culture

Cells were isolated by using a previously described with modification with minor modifications [21]. In brief, bone marrow samples diluted 1:1 with DPBS were layered on Ficoll-Paque Premium (GE Healthcare, Uppsala, Sweden) and centrifuged at 400 g (brake off) for 30 min at 20 °C. The top layer was discarded, and the mononuclear cell layer was collected and diluted with three volumes of DPBS. This suspension was centrifuged again at 400 g for 5 min, then washed with DPBS. The cell pellet was resuspended in LG-DMEM supplemented with 10% FBS and antibiotic–antimycotic solution, and plated at a density of 1 × 10^5^ cells/cm^2^ in a 5% CO_2_ incubator with humidified air at 37 °C. The culture medium was changed every 2–3 days. When the cells reached 80% confluency, they were detached using a 3-min incubation with trypsin–EDTA, washed, and then split at a 1:4 ratio. BM MSCs at passage four to five were used.

### AD MSCs isolation and culture

Adipose tissue was obtained through tissue aspiration and extensively washed with equal volumes of DPBS to remove blood products. The tissue was finely minced using surgical scissors and scalpels. Cells were released by treating the tissue with 0.1% collagenase type I for 1 h at 37 °C with gentle agitation. The collagenase was then inactivated with an equal volume of DPBS, and the suspension was centrifuged at 1,200 g for 10 min at 20 °C. The pellet was resuspended in DPBS and filtered through a 100 µm cell strainer to remove debris. The filtered cells were centrifuged again, resuspended in LG-DMEM supplemented with 10% FBS and an antibiotic–antimycotic solution, and plated at a density of 5 × 10^3^ cells/cm^2^ in a 5% CO_2_ incubator with humidified air at 37 °C. The culture medium was changed every 2–3 days. When the cells reached 80% confluency, they were detached using a 3-min incubation with trypsin–EDTA, washed, and then split at a 1:3 ratio. AD MSCs at passage four to five were used.

### Total RNA isolation

Total RNA was extracted using the TRIzol® RNA Isolation Reagents (Life Technologies, Korea). The RNA quantity and quality were assessed with an Agilent 2100 Bioanalyzer RNA kit (Agilent). The extracted RNA was then used to prepare the mRNA sequencing library with the Illumina TruSeq Stranded mRNA Sample Preparation kit (Illumina, US) following the manufacturer’s instructions. The quality and size of the libraries were checked using the Agilent 2100 Bioanalyzer DNA kit (Agilent Technologies, US). All libraries were quantified by qPCR on a CFX96 Real-Time System (Bio-Rad) and sequenced on the NextSeq500 sequencer (Illumina, US) with a paired-end 75 bp plus single 8 bp index read run. OD measurements were performed using the Dropsense96 system (Trinean, Belgium). Quality checks were conducted with the Bioanalyzer RNA Chip (Agilent Technologies, US). Fluorescence quantification was carried out using the Ribogreen assay (Quant-it™ RiboGreen RNA Assay Kit, Invitrogen, US).

### RNA sequencing

Raw reads sequenced from samples were processed to generate a gene count matrix using the following steps: First, Trimmomatic (v0.39) was employed to remove adaptor sequences [22]. Subsequently, the trimmed reads were mapped to the human reference genome (hg38) using STAR (v2.7.8a) [23]. The aligned reads were sorted using samtools (v1.9) [24]. Finally, gene counts were determined using HTSeq (v2.0.4) [25]. Genes with a sum of counts across samples less than 2 were excluded. The final gene count matrix comprised 28,703 genes and 15 samples.

### Identification of differentially expressed genes (DEGs)

To identify differentially expressed genes (DEGs) among groups, we employed DESeq2 (v1.26.0), utilizing the Wald test for two-group comparisons and the likelihood ratio test (LRT) for comparing more than two groups were used [26]. Default values were maintained for other options. DEGs across all groups were initially identified at a false discovery rate (FDR) 0.05. In the two-group comparisons, genes were considered significant with an FDR less than 0.05 and a log2 fold change (log_2_FC) greater than 0.5 or less than -0.5. Among these genes, only those included in DEGs across all groups were regarded as DEGs in that specific comparison to minimize false positives.

### Identification of pathways and transcription factors associated with DEGs

The DEGs identified in each comparison were further analyzed to determine the pathways or transcription factors associated with them. For this analysis, we utilized the MSigDB Hallmark 2020, TRRUST Transcription Factors 2019, and GO Biological Process 2023 databases implemented in Enrich R (https://maayanlab.cloud/Enrichr/) [27]. The analyses aimed to identify the top 10 terms significantly associated with upregulated or downregulated DEGs at FDR 0.05. The FDRs of these associations were then compared with those of DEGs in the opposite direction on a -log10 scale.

### Clustering analysis

The count values were transformed into log counts per million (logcpm). Subsequently, sparse principal components were estimated for all samples using the *sparsepca* (v0.1.2) R package [28]. In each two-group comparison, groups were divided into clusters based on their top 20 upregulated and downregulated DEGs. The results were visually represented as heatmaps using the *pheatmap* (v1.0.12) R package [29]. Additionally, heatmaps were drawn for up to 20 DEGs included in gene lists of selected gene ontology (GO) terms. The gene lists corresponding to the GO terms were obtained from https://amigo.geneontology.org [30].

### Module score analysis

We computed gene set activity scores in the form of module scores. Using genes, not limited to DEGs, included in a specific term, the scores were calculated. The terms were selected based on the MSigDB Hallmark 2020 pathways in which the identified DEGs were enriched, and their gene lists were obtained from https://www.gsea-msigdb.org/gsea/msigdb. A Seurat object was created, normalized, and then module scores were computed using the AddModuleScore function in the Seurat (v4.3.0.1) R package [31]. Linear regression was employed to compare module scores among groups. All analyses were conducted in the R environment (v3.6.3) after generating the gene count matrix. For visualization including pca plots, bar plots, box plots, and volcano plots, ggplot2 R package were used [32].

## Results

### RNA sequencing data analysis

**Fig. 1:**
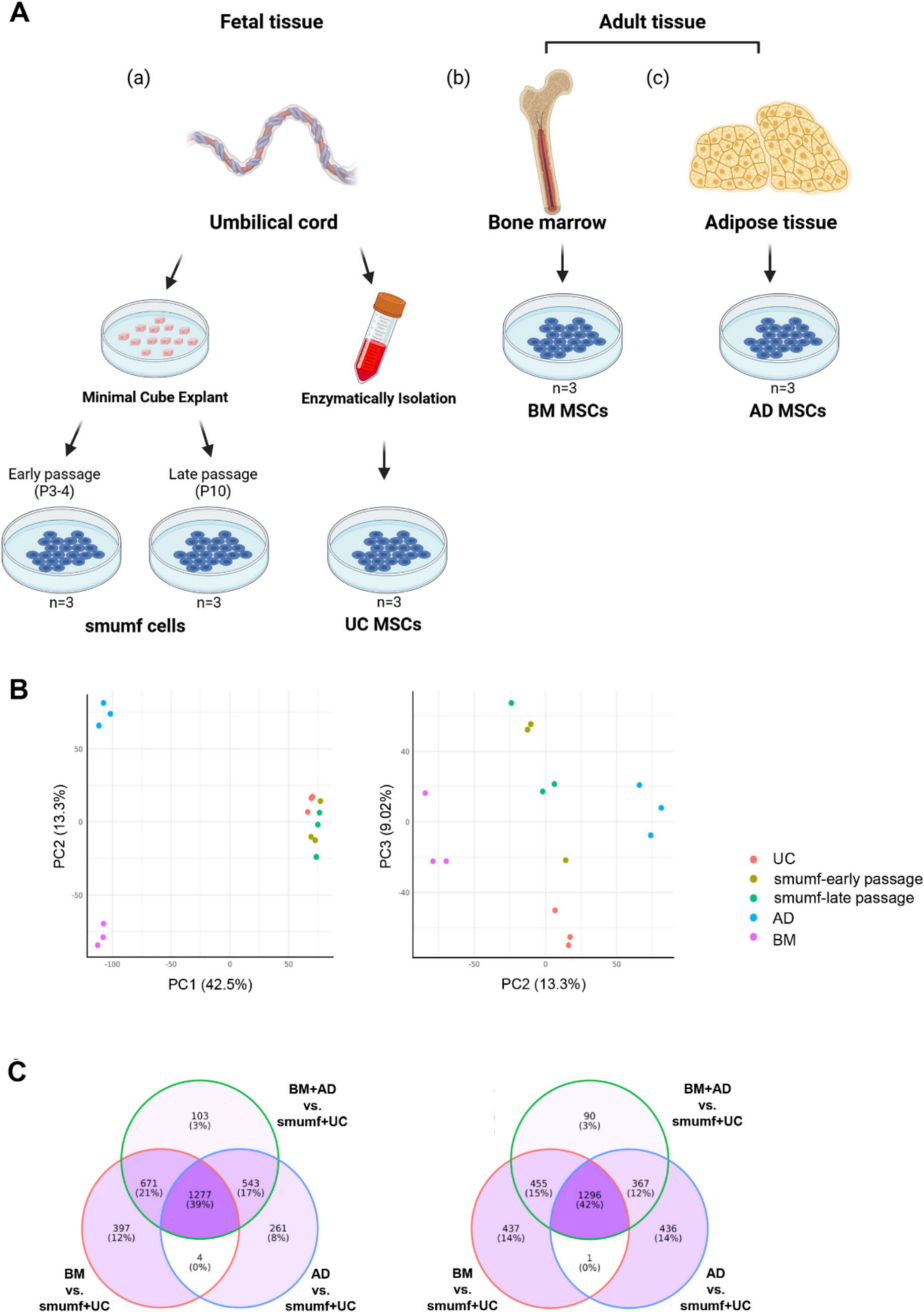
RNA-seq data analysis of UC, smumf, AD, and BM-MSCs. **A** Isolation scheme of MSCs. **B** Sparse PCA plots displaying top 3 PC scores AD, BM, smumf-early, smumf-late, and UC MSCs. The percentage of variance explained by each component is indicated on the axes. Left and right panels show PC1 and 2, and PC2 and 3, respectively. **C** Venn Diagrams of DEGs in three models. Venn diagrams depicting upregulated (left panel) and downregulated (right panel) DEGs in BM + AD, BM, and AD MSCs compared to fetal MSCs

When comparing fetal MSCs with AD and BM, respectively, we observed similar patterns, as seen when comparing them (Fig. 1 and supplementary table S1). For AD, out of 2,085 upregulated and 2,100 downregulated DEGs compared to fetal MSCs, 1,820 (87%) and 1,663 (79%) genes are common when comparing fetal MSCs and both types of adult MSCs. In the case of BM, out of 2,349 upregulated and 2,189 downregulated DEGs compared to fetal MSCs, 1,948 (83%) and 1,751 DEGs (80%) were common when comparing fetal MSCs and both types of adult MSCs.

### Distinct gene expression patterns among MSCs

**Fig. 2:**
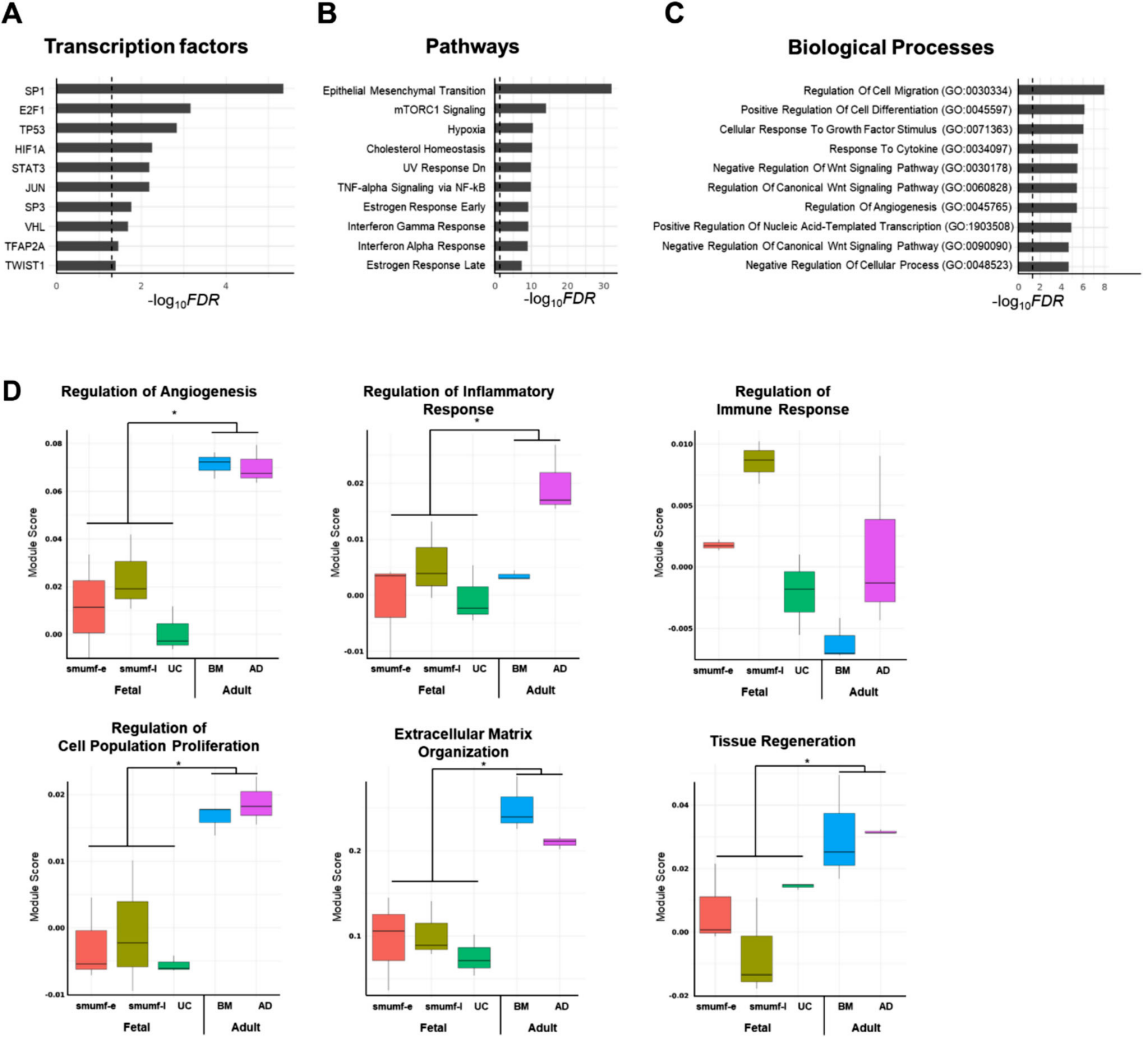

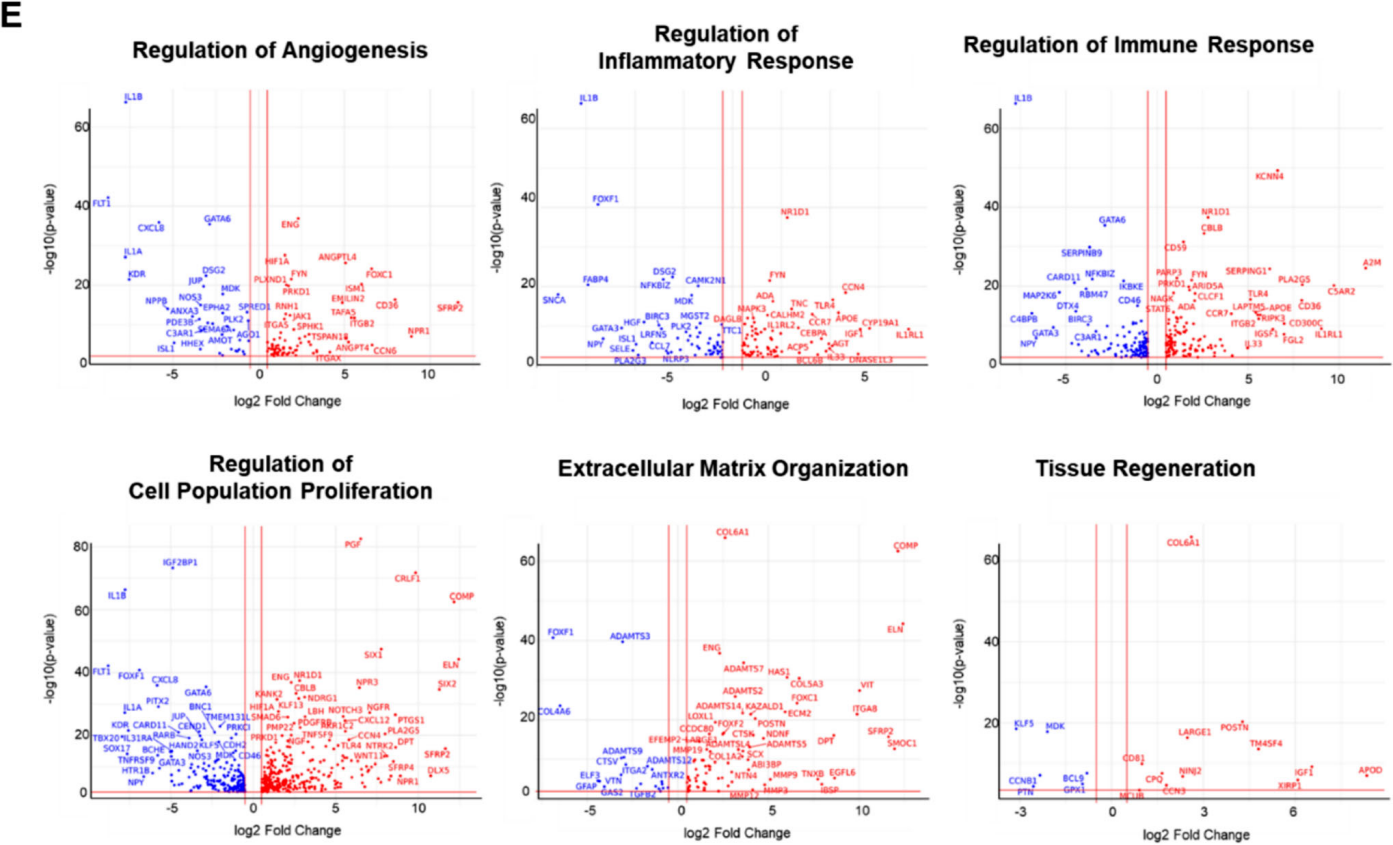
Comparison among MSCs. (A-C) Bar plots showing enrichment analysis of **A** transcription factors, **B** pathways, and **C** biological processes associated with differentially expressed genes (DEGs) across five mesenchymal stem cell (MSC) types (smumf early and late, UC, BM, and AD). (D) Boxplots comparing module scores of selected biological processes across MSC types. Five comparisons were tested: (1) all-group comparison, (2) fetal (smumf and UC) vs. adult (BM and AD), (3) smumf vs. UC, (4) early vs. late passages of smumf, and (5) BM vs. AD. Statistically significant comparison are labeled with asterisks at a nominal p-value of 0.05. (**E**) Volcano plots of DEGs in selected pathways comparing fetal and adult MSCs. (A-C) The x-axis represents –log_10_(FDR), the y-axis displays top 10 transcription factors, pathways, or biological processes, and the vertical dashed line marks − log_10_(0.05). **D** The x-axis represents types of MSCs and the y-axis shows the estimated values of module scores. **E** The x-axis shows log_2_ fold changes and the y-axis shows -log_10_(p-value). The red horizontal lines indicate the p-value threshold corresponding approximately to a false discovery rate of 0.05, and the red vertical lines represent log_2_ fold changes of 0.5 and -0.5. Red and blue dots indicate genes highly expressed in adult and fetal MSCs, respectively. FDR; false discovery rate

We identified a total of 8,091 DEGs across five MSC types (smumf early and late, UC, BM, and AD). Enrichment analysis revealed that these genes were significantly associated with transcription factors such as SP1, E2F1, TP53, HIF1A, and STAT3 (Fig. 2A); pathways including epithelial mesenchymal transition, mTORC1 signaling, hypoxia, cholesterol homeostasis, and UV response down (Fig. 2B); and biological process including regulation of cell migration, positive regulation of cell differentiation, cellular response to growth factor stimulus, response to cytokine, negative regulation of Wnt signaling pathway (Fig. 2C).

In particular, we focused on pathways related to angiogenesis, inflammatory response, immune response, cell proliferation, extracellular matrix (ECM) organization, and tissue regeneration. Module score comparisons showed significant differences between fetal (smumf and UC) and adult (BM and AD) MSCs in all categories except for “regulation of inflammatory response” and “regulation of immune response” at FDR 0.05 (Fig. 2D). The former showed significant differences between BM and AD MSCs, while the latter varied significantly among fetal MSCs. Notably, *IL1B*, a gene involved in multiple biological processes (angiogenesis, inflammation, immune modulation, and proliferation), showed one of the highest log_2_ fold changes between fetal and adult MSCs, being highly expressed in fetal MSCs (Fig. 2E). For ECM related genes, *FOXF1*, *COL4A6*, *ADAMTS3* and *ADAMTS9* were highly expressed in fetal MSCs, while *COL6A1*, *COMP*, and *ELN* were predominant in adult MSCs. For tissue regeneration, genes such as *KLF5*, *MDK*, and *CCNB1* were enriched in fetal MSCs, whereas *COL6A1*, *POSTN*, *APOD*, and *IGF1* were enriched in adult MSCs. Several DEGs involved in these biological processes were also observed in comparisons between smumf and UC MSCs, between early and late passages of smumf MSCs, and between BM and AD MSCs (Supplementary Fig. 2). However, while module scores for immune regulation differed significantly between early and late passage smumf cells, no DEGs in that pathway met the FDR threshold.

### Distinct gene expression patterns in fetal vs. adult MSCs

To compare the transcriptomic profiles of MSCs derived from fetal and adult tissues, UC and smumf (from early and late passages) were compared with AD and BM. Gene expression levels were analyzed among 4 types of MSCs: AD, BM, UC, and smumf. AD, BM, and UC each included 3 samples, while smumf comprised early and late passage samples, with three replicates each (Fig. 1A). Clustering of samples based on their complete transcriptomic data and principal component (PC) scores revealed distinct groupings for AD, BM, UC, and smumf cells (Fig. 1B). Each type displays a distinct gene expression pattern compared to the others. Notably, fetal MSCs (UC and smumf) exhibited greater proximity to each other than adult MSCs (AD and BM).

Initially, we identified differentially expressed genes (DEGs) between fetal and adult MSCs. In fetal MSCs, we found 2,208 upregulated and 2,594 downregulated DEGs at a false discovery rate (FDR) 0.05, satisfying an absolute log_2_FC greater than 0.5 (Fig. 1C). Among the upregulated DEGs in fetal MSCs are *BDKRB1*, *MYRF*, *SHC3*, *DSC3*, and *IGF2BP1*, while the downregulated DEGs include *ENPP2*, *PDE1C*, *CYGB*, *PGF*, and *TBX15* (Table 1).

**Table 1:** Top 10 DEGs upregulated and downregulated in fetal MSCs compared to adult MSCs, meeting criteria of log_2_|FC|> 0.5 and FDR < 0.05. DEGs, differentially expressed genes; MSCs, mesenchymal stem cells; FC, fold change; FDR, false discovery rate

Top DEGs in fetal MSCs compared to adults MSCs				
Gene	Description	Log_2_lFCl	P-value	FDR
*Upregulated DEGs*				
BDKRB1	Bradykinin receptor B1	6.266	6.54E-192	1.87E-187
MYRF	Myelin regulatory factor	7.411	1.73E-173	2.47E-169
SHC3	SHC adaptor protein 3	5.577	6.84E-100	4.89E-96
DSC3	Desmocollin 3	10.010	8.61E-94	4.93E-90
IGF2BP1	Insulin like growth factor 2 mRNA binding protein 1	4.876	5.15E-74	1.47E-70
RTN1	Reticulon 1	6.514	8.74E-68	1.79E-64
ENSG00000224078	Small nucleolar RNA host gene 14	1.709	1.35E-67	2.58E-64
SORL1	Sortilin related receptor 1	5.929	2.14E-67	3.82E-64
IL1B	Interleukin 1 beta	7.757	4.54E-67	7.22E-64
ST6GALNAC5	ST6 N-acetylgalactosaminide alpha-2,6-sialyltransferase 5	9.426	2.33E-65	3.17E-62
*Downregulated DEGs*				
ENPP2	Ectonucleotide pyrophosphatase/phosphodiesterase 2	7.830	6.10E-102	5.82E-98
PDE1C	Phosphodiesterase 1C	10.266	4.75E-90	2.26E-86
CYGB	Cytoglobin	7.170	1.28E-83	5.25E-80
PGF	Placental growth factor	6.505	2.76E-83	9.87E-80
TBX15	T-box transcription factor 15	12.877	7.95E-78	2.53E-74
COL6A2	Collagen type VI alpha 2 chain	3.109	1.13E-73	2.95E-70
CRLF1	Cytokine receptor like factor 1	9.832	1.89E-72	4.49E-69
ACAN	Aggrecan	9.972	3.31E-69	7.27E-66
LSP1	Lymphocyte specific protein 1	9.636	3.26E-67	5.48E-64
COL6A1	Collagen type VI alpha 1 chain	2.605	1.18E-66	1.77E-63

**Fig. 3:**
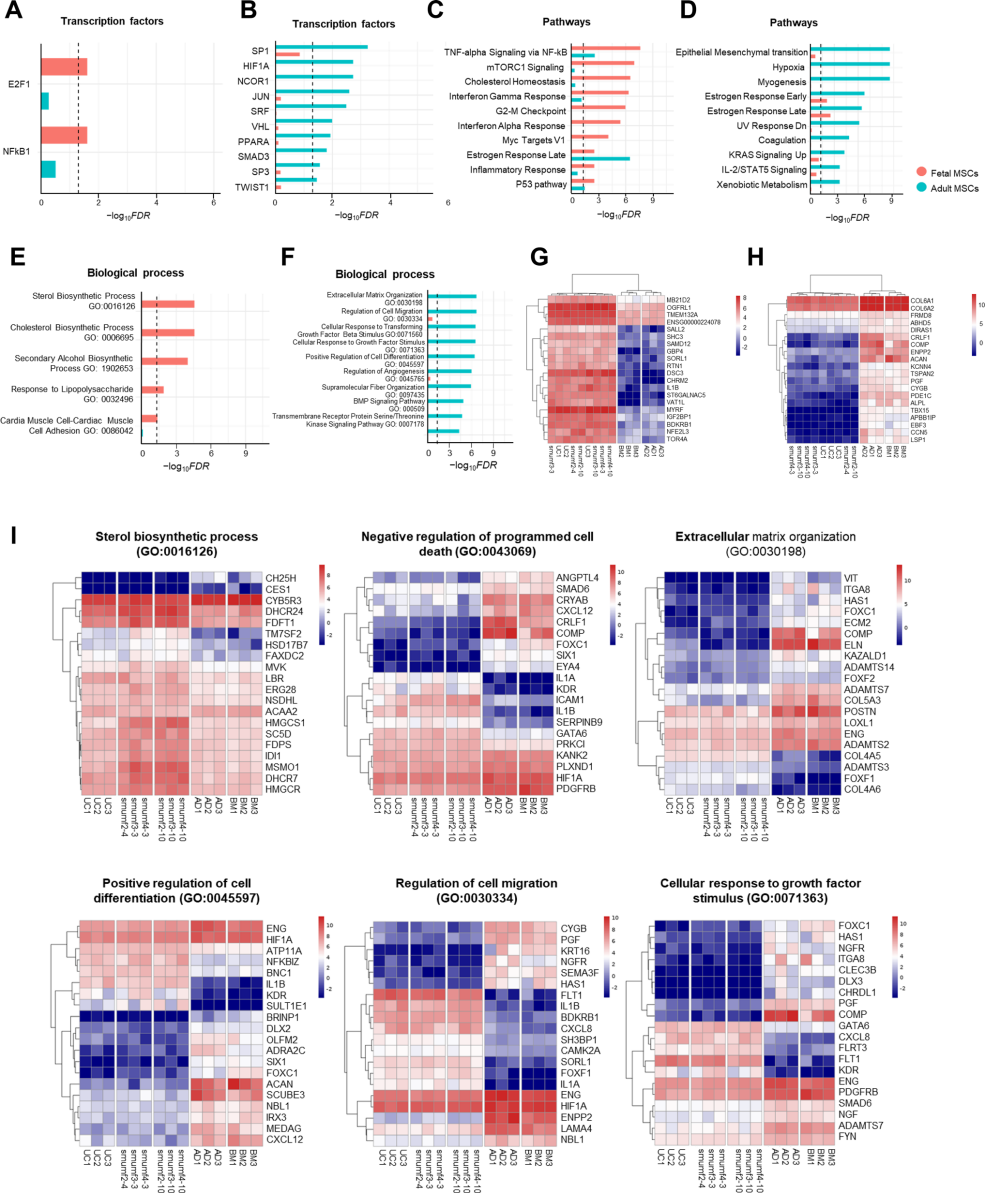
Comparison between fetal and adult MSCs. (A-B) Barplots for transcription factors significantly associated with **A** up- and **B** down-regulated genes between fetal and adults MSCs at FDR 0.05. (C-D) Barplots for pathways significantly associated with **C** up- and **D** down-regulated genes between fetal and adults MSCs at FDR 0.05. (E–F) Gene Ontology enrichment analysis of biological processes for **E** up- and **F** down-regulated genes between fetal and adult MSCs. (A-F) x-axis shows –log_10_(FDR), y-axis displays maximum top 10 transcription factors or pathways, and dotted vertical line represents − log_10_(0.05). Red and turquoise indicate the –log_10_(FDR) of transcription factors or pathways in which upregulated and downregulated DEGs in fetal MSCs were enriched, respectively. (G-H) Heatmaps of top 20 **G** up- and **H** down-regulated DEGs in fetal MSCs. **I** Heatmaps depicting significant Gene Ontology enrichment analysis of individual biological processes. TFs; transcription factors, FDR; false discovery rate, MSCs; mesenchymal stem cells

Transcription factor analysis reveals significant associations of upregulated DEGs in fetal MSCs with E2F1 and NF-κB1 transcription factors (TFs) (Fig. 3A). Conversely, downregulated DEGs in fetal MSCs are significantly associated with SP family (SP1 and SP3), HIF1A, PPARA, SMAD3, and TWIST1 TFs (Fig. 3B). Pathway analysis identifies enriched pathways in upregulated DEGs, such as TNF-alpha signaling via NF-kB, mTORC1 signaling, cholesterol homeostasis, interferon alpha and gamma response, and G2-M checkpoint (Fig. 3C). Downregulated DEGs are significantly associated with pathways including epithelial-mesenchymal transition, hypoxia, KRAS signaling up, IL-2/STAT5 signaling, and xenobiotic metabolism (Fig. 3D).

Biological process analysis highlights significant associations of upregulated DEGs in fetal MSCs with sterol biosynthetic processes, cholesterol biosynthetic processes, secondary alcohol biosynthetic processes, and responses to lipopolysaccharides (Fig. 3E). These processes suggest a strong metabolic and biosynthetic activity in fetal MSCs. Conversely, downregulated DEGs in fetal MSCs are significantly enriched in biological processes such as extracellular matrix organization, regulation of cell migration, cellular response to growth factor stimuli, positive regulation of cell differentiation, regulation of angiogenesis, and BMP signaling pathways (Fig. 3F), emphasizing the structural and regenerative roles more pronounced in adult MSCs.

Heatmap clustering of the top DEGs provided a clear visualization of the distinctions between fetal and adult MSCs (Fig. 3G and H). Notably, fetal MSCs (UC and smumf) show elevated expression of genes such as *MB21D2* and *OGFRL1*, which are involved in metabolic regulation and developmental pathways. In contrast, adult MSCs (AD and BM) exhibit significantly higher expression of *COL6A1*, *COL6A2*, and *COMP*, which are crucial for ECM structure and integrity. Heatmaps further emphasize these differences, highlighting genes enriched in the sterol biosynthetic process (GO:0016126), negative regulation of programmed cell death (GO:0043069), extracellular matrix organization (GO:0030198), positive regulation of cell differentiation (GO:0045597), regulation of cell migration (GO:0030334), and cellular response to growth factor stimulus (GO:0071363) (Fig. 3I).

### Distinct gene expression patterns in smumf vs. UC MSCs

**Fig. 4:**
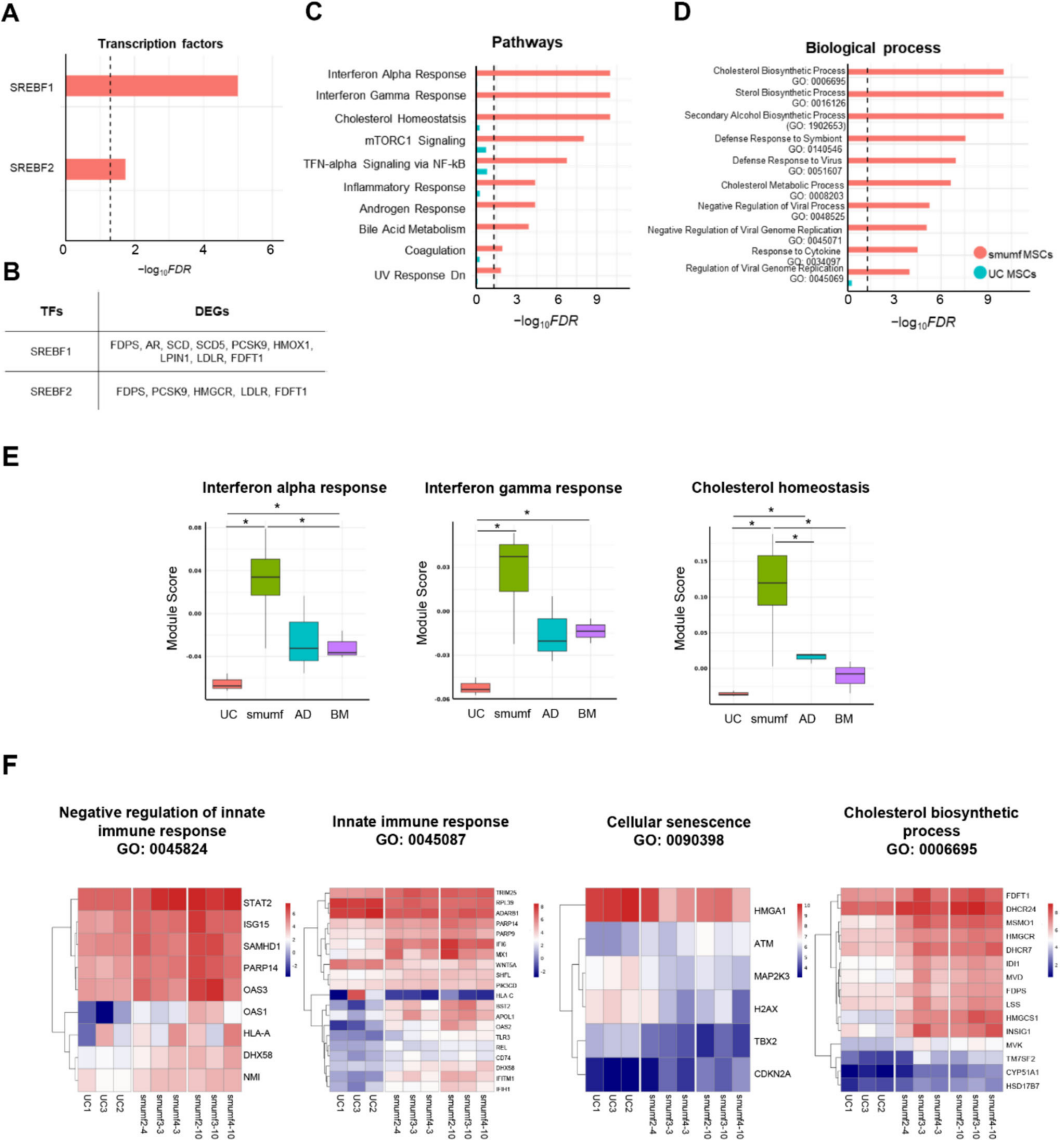
Comparison between smumf and UC MSCs. **A** Barplot illustrating transcription factors significantly associated with upregulated DEGs in smumf cells compared to UC MSCs at FDR 0.05. **B** Transcription factors and genes regulated by each among the upregulated DEGs in smumf MSCs. (C-D) Barplots representing **C** pathways and **D** biological processes in which upregulated DEGs in smumf cells are enriched compared to UC MSCs at FDR 0.05. **E** Boxplots displaying module scores of pathways across MSC type. The asterisk indicates that the module scores of the two groups are significantly different at a nominal p-value of 0.05. (A,C-D) x-axis shows –log_10_(FDR), y-axis displays maximum top 10 transcription factors or pathways, and dotted vertical lines represent − log_10_(0.05). Red and turquoise indicate –log_10_(FDR) of corresponding transcription factors or pathways for upregulated and downregulated DEGs in smumf cells, respectively. **F** Heatmaps depicting significant biological processes from Gene Ontology. TFs; transcription factors, FDR; false discovery rate, MSCs; mesenchymal stem cells, DEGs; differentially expressed genes

To determine the difference between UC MSCs isolated by the explant (smumf) method and the enzymatic (UC MSCs) method, distinct gene expression patterns were observed. The comparison of smumf cells and UC MSCs identified 644 upregulated and 545 downregulated DEGs in smumf cells in comparison to UC (Table 2). Transcription factor analysis highlights significant associations of SREBF1 and SREBF2 with the upregulated DEGs in smumf MSCs (Fig. 4A), including *FDPS*, *PCSK9*, *LDLR*, and *FDFT1* (Fig. 4B). Pathway analysis significantly links upregulated DEGs in smumf MSCs to innate immune responses (interferon alpha response, interferon gamma response, and TNF-alpha signaling via NF-kB pathway), metabolism processes (cholesterol homeostasis and bile acid metabolism), and cell cycle pathways (mTORC1 signaling) (Fig. 4C). However, downregulated DEGs in smumf cells do not show any significantly associated TFs or pathways.

**Table 2:** Top 10 DEGs upregulated and downregulated in smumf cells compared to UC MSCs, meeting criteria of log_2_|FC|> 0.5 and FDR < 0.05. DEGs, differentially expressed genes; MSCs, mesenchymal stem cells; FC, fold change; FDR, false discovery rate

Top DEGs in smumf cells compared to UC MSCs				
Gene	Description	Log_2_lFCl	P-value	FDR
*Upregulated DEGs*				
RPL26	ribosomal protein L26	2.801	1.75E-70	2.44E-66
TAX1BP3	Tax1 binding protein 3	2.657	4.74E-40	3.30E-36
ATP8	mitochondrially encoded ATP synthase membrane subunit 8	3.193	1.09E-39	5.21E-36
TOMM5	translocase of outer mitochondrial membrane 5	3.501	1.07E-27	2.30E-24
PSMA6	proteasome 20S subunit alpha 6	2.290	1.00E-23	1.64E-20
MICOS10	mitochondrial contact site and cristae organizing system subunit 10	1.975	9.57E-23	1.48E-19
HMGCS1	3-hydroxy-3-methylglutaryl-CoA synthase 1	3.290	6.82E-21	7.39E-18
ENSG00000210135	mitochondrially encoded tRNA-Asn (AAU/C)	10.984	1.60E-19	1.39E-16
MSMO1	methylsterol monooxygenase 1	2.389	7.91E-19	6.67E-16
IFITM1	interferon induced transmembrane protein 1	3.747	2.20E-17	1.61E-14
*Downregulated DEGs*				
H3-3A	H3.3 histone A	2.267	6.08E-82	1.69E-77
ENSG00000233476	eukaryotic translation elongation factor 1 alpha 1 pseudogene 6	3.123	3.21E-47	2.98E-43
ENSG00000186076	phosphoglycerate mutase 1 (brain) (PGAM1) pseudogene	3.727	1.12E-39	5.21E-36
ENSG00000250182	eukaryotic translation elongation factor 1 alpha 1 pseudogene 13	2.473	6.26E-37	2.49E-33
ENSG00000249264	eukaryotic translation elongation factor 1 alpha 1 pseudogene 9	3.508	4.22E-36	1.47E-32
ENSG00000247627	MT-ND4 pseudogene 12	3.569	1.88E-34	5.81E-31
POTEE	POTE ankyrin domain family member E	5.158	1.36E-30	3.79E-27
EIF3CL	eukaryotic translation initiation factor 3 subunit C like	4.352	1.10E-28	2.78E-25
ENSG00000235552	ribosomal protein L6 pseudogene 27	2.335	1.68E-28	3.89E-25
MTRNR2L12	MT-RNR2 like 12	4.921	1.32E-26	2.62E-23

Module score analysis reveals significantly higher innate immune responses, including interferon alpha and gamma responses, as well as cholesterol homeostasis in smumf MSCs compared to other MSC types (Fig. 3E). Heatmap analysis further emphasizes these differences, highlighting enriched expression of genes involved in the negative regulation of innate immune response (GO:0045824), innate immune response (GO:0045087), cellular senescence (GO:0090398), and cholesterol biosynthetic processes (GO:0006695) in smumf MSCs compared to UC MSCs (Fig. 3F). The data exhibit distinct the metabolic flexibility and immune readiness of smumf MSCs, as reflected by their elevated module scores for cholesterol biosynthesis and innate immune regulation pathways.

### Gene expression patterns in early vs. late passages of smumf MSCs

**Fig. 5:**
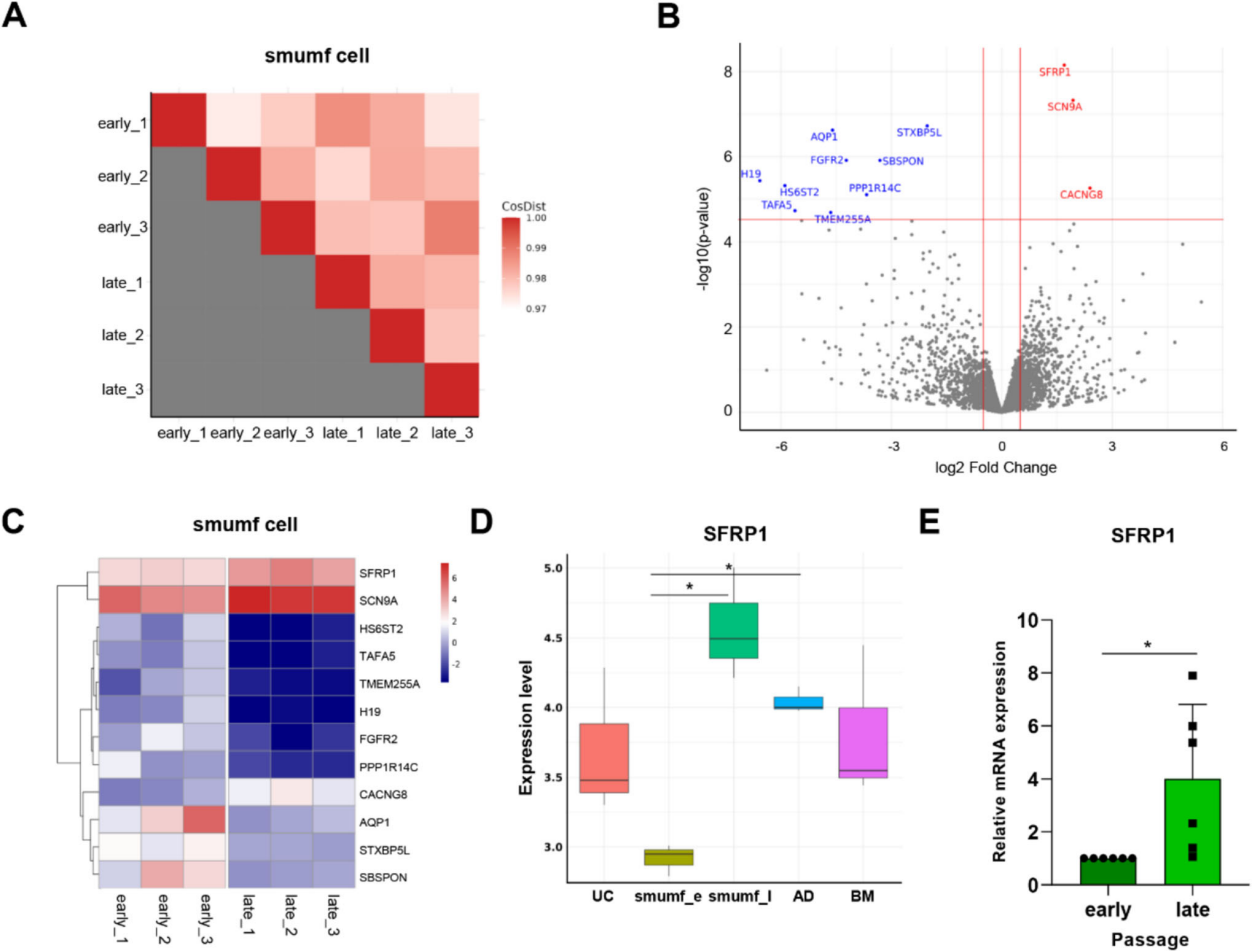
Comparison between early and late passages of smumf cells. **A** Tile plot for cosine distance demonstrated similarity of genes expression between early and late passages of smumf MSCs. **B** Volcano plot showing log_2_ fold change and -log_10_ p-values of gene expression between early and late passages. The red horizontal line indicates the significance threshold (-log_10_($3\times {10}^{-5}$), corresponding approximately to a false discovery rate of 0.05), and the red vertical lines represent log_2_ fold changes of 0.5 and -0.5, respectively. Red and blue dots indicate DEGs significantly up-regulated in late and early passages, respectively. **C** Heatmaps depicting 12 DEGs across groups. **D** Boxplot displaying expression levels of *SFRP1* gene across MSC type. Each box represents inter quantile range (IQR) from Q1 to Q3 with bold line median value, and the whiskers show values within Q3 + 1.5 $\times$ IQR and Q1-1.5 $\times$ IQR. **E** mRNA expression of *SFRP1* in smumf_e and smumf_l. The asterisk indicates that the gene expression levels of the two groups are significantly different at a nominal p-value of 0.05 (n = 6). CosDist, cosine distance; smumf_e, early passage of smumf MSCs; smumf_l, late passage of smumf MSCs

When comparing the gene expression profiles of early and late passages of smumf cells, only 12 genes displayed significant differences between the two groups. The heatmap, depicting cosine distance or Pearson’s correlation, revealed a similarity, with a value nearly approaching 1.00 (Fig. 5A and Supplementary Fig. 3). This observation strongly suggests that the expression levels of genes in late passage smumf MSCs closely resembles those of early passage MSCs, indicating that late-passage MSCs have retained the gene characteristics of their early-passage counterparts.

We identify only 9 upregulated and 3 downregulated DEGs in the early passage compared to the late passage of smumf MSCs (Table 3 and Fig. 5B–C). Among these, *SFRP1* is notably the most significantly different gene, exhibiting higher expression in the late passage than in the early passage (Fig. 4D). The elevated expression level of *SFRP1* in the late passage compared to the early passage was further confirmed by real-time PCR (Fig. 4E).

**Table 3:** DEGs upregulated and downregulated in early passage of smumf cells compared to late passage of smumf cells, meeting criteria of log_2_|FC|> 0.5 and FDR < 0.05. DEGs, differentially expressed genes; MSCs, mesenchymal stem cells; FC, fold change; FDR, false discovery rate

Top DEGs in the early passage of smumf compared to the late passage of smumf cells				
Gene	Description	Log_2_lFCl	P-value	FDR
*Upregulated DEGs*				
STXBP5L	syntaxin binding protein 5 like	2.027	1.91E-07	0.002
AQP1	aquaporin 1 (Colton blood group)	4.601	2.39E-07	0.002
FGFR2	fibroblast growth factor receptor 2	4.227	1.23E-06	0.006
SBSPON	somatomedin B and thrombospondin type 1 domain containing	3.313	1.23E-06	0.006
H19	H19 imprinted maternally expressed transcript	6.577	3.71E-06	0.014
HS6ST2	heparan sulfate 6-O-sulfotransferase 2	5.895	4.84E-06	0.015
PPP1R14C	protein phosphatase 1 regulatory inhibitor subunit 14C	3.673	7.89E-06	0.020
TAFA5	TAFA chemokine like family member 5	5.620	1.85E-05	0.042
TMEM255A	transmembrane protein 255A	4.651	2.04E-05	0.043
*Downregulated DEGs*				
SFRP1	secreted frizzled related protein 1	1.697	7.21E-09	0.000
SCN9A	sodium voltage-gated channel alpha subunit 9	1.935	4.74E-08	0.001
CACNG8	calcium voltage-gated channel auxiliary subunit gamma 8	2.397	5.53E-06	0.015

### Distinct gene expression patterns in AD vs. BM MSCs

Finally, we compared gene expression levels between 2 types of adult MSCs: AD and BM. A total of 947 are significantly upregulated in AD, while 918 genes show significant upregulation in BM (Supplementary table S2). For instance, AD exhibits upregulation in genes such as *IGFBP2*, *SRGN*, *PLCB4*, *EDIL3*, and *IGF2*, while BM shows upregulation in *IL13RA2*, *SFRP4*, *PI16*, *PKDCC*, and *EPB41L3*. The upregulated DEGs in AD are significantly enriched in H1F1A and SP1 TFs (Supplementary fig. S1A). In contrast, H1F1A is not significantly associated with upregulated DEGs in BM, whereas E2F1, TP53, and TWIST1 TFs are exclusively and significantly associated with upregulated DEGs in BM (Supplementary fig. S1B). Several pathways demonstrate shared significance with upregulated DEGs in both AD and BM, such as epithelial-mesenchymal transition and hypoxia (Supplementary fig. S1C). Module scores of these pathways are higher in adult MSCs than in fetal MSCs (Supplementary fig. S1D). Some biological processes highlight differences between the two MSC types. Upregulated DEGs in AD-MSCs are linked to endodermal cell differentiation and endoderm formation (Supplementary fig. S1E), while those in BM-MSCs are associated with energy metabolism, including the negative regulation of macromolecule metabolic process and GMP metabolic process (Supplementary fig. S1F).

## Discussion

The most important findings of this study are (1) our study confirmed that smumf cells maintain a consistent transcriptome profile, with only 12 differentially expressed genes (DEGs) out of 28,703 (0.04%) from early (P3-4) to late (P10) passages. (2) We identified key transcription factors significantly associated with elevated DEGs in fetal MSCs compared to adult MSCs, with E2F1 and NF-κB1 showing strong associations, which may contribute to the superior immunomodulatory and regenerative potential of fetal MSCs. (3) Significant upregulation of glycolysis was observed in fetal MSCs, particularly in smumf cells, which also exhibited higher expression of genes related to cholesterol and sterol biosynthesis, and the interferon and TNF-α signaling pathways. Taken together, these findings emphasize the distinct RNA profile of fetal MSCs compared to adult MSCs, providing deeper insights into their immunomodulatory and regenerative potential for therapeutic applications.

Fetal MSCs, derived from the umbilical cord (UC), umbilical cord blood (UCB), and placenta, exhibit superior characteristics compared to adult MSCs sourced from tissues such as bone marrow (BM) and adipose tissue (AD) [6, 18, 33]. These advantages include higher proliferation rates, greater differentiation potential, and lower immunogenicity [6, 7]. Such properties are particularly valuable for clinical applications, as fetal MSCs retain their stemness and regenerative capacity more effectively than adult MSCs, which often experience age-related declines in these functions [10, 11]. One key benefit of fetal MSCs is their ability to maintain high proliferation rates, enabling efficient expansion to clinical quantities without compromising functional integrity. For instance, UC MSCs exhibit a faster proliferative capacity, allowing expansion to higher passages while maintaining sufficient cell numbers. In contrast, BM MSCs face limitations due to their invasive collection methods and a progressive decline in proliferation potential with donor age. This distinction underscores the practical and biological advantages of fetal MSCs in regenerative medicine [13, 14]. Additionally, fetal MSCs exhibit enhanced immunomodulatory properties through the secretion of higher concentrations of cytokines, such as IL-10, IL-8, TGF-β2, and HGF, which play crucial roles in suppressing T-cell proliferation and modulating immune responses [34]. These cytokines also contribute to tissue repair and inflammation regulation, positioning fetal MSCs as promising therapeutic candidates for autoimmune diseases, tissue regeneration, and cancer therapy [8, 9]. Furthermore, fetal MSCs are less susceptible to DNA damage caused by environmental stressors, a common challenge in adult stem cells, which enhances their suitability for long-term clinical applications [12].

Expanding MSC cultures while maintaining their stem cell properties is critical to achieving the large quantities needed for clinical applications. While previous studies have highlighted the challenges of prolonged culture, such as replicative exhaustion and transcriptional changes, our findings provide further evidence of the stability of UC MSCs during extended passages. Earlier research demonstrated that UC MSCs retain high expression of stem cell markers, such as NANOG, DNMT3B, and GABRB3, and pluripotent markers even at late passages [35]. Additionally, Wiese et al. observed that transcriptional profiles of UC MSCs remained relatively stable through pre-senescence stages (P1 to P12) [36]. In line with these findings, our study confirmed that smumf cells maintain a consistent transcriptome profile, with only 12 differentially expressed genes (DEGs) out of 28,703 (0.04%) from early (P3-4) to late (P10) passages. This minimal variation underscores the transcriptional stability of smumf cells during long-term culture. Among the DEGs, Secreted Frizzled-Related Protein-1 (SFRP1) was significantly upregulated in late-passage UC MSCs. As a modulator of Wnt signaling, SFRP1 plays a critical role in tissue repair and proliferation. Previous studies suggest that SFRP1 enhances MSC function, including angiogenesis and neovessel maturation, and may contribute to maintaining cell proliferation during extended culture [37, 38]. In our previous study, no copy number variations (CNVs) were observed between smumf cells from P3 to P10, indicating sustained genomic stability following serial passaging. Furthermore, the consistent expression of markers such as p16, p21, and p53, along with the lack of a significant increase in SA-β-gal activity up to P10, highlights their resistance to senescence compared to BM MSCs [18, 20]. Taken together, these findings underscore the suitability of UC MSCs, particularly smumf cells, as a reliable and stable cell source for therapeutic applications that demand large-scale expansion, while maintaining their stemness, proliferative capacity, and functional integrity during extended *in vitro* culture.

Fetal MSCs exhibit enhanced immunomodulatory properties through the secretion of higher concentrations of cytokines compared to adult MSCs [34]. Our study identified key transcription factors that are significantly elevated in fetal MSCs compared to adult MSCs, with E2F1 and NF-κB1 showing a significant increase. E2F is a key regulator of cell cycle progression and is controlled by pRb, which regulates E2F activity [39]. Since NF-κB activation in primary cells, such as chondrocytes and tenocytes, is largely detrimental due to its role in promoting inflammation, NF-κB activation in MSCs can be beneficial as it enhances immunomodulation, regulates differentiation, and inhibits osteogenesis [40–42]. NF-κB has been shown to induce the migration and proliferation of human MSCs in response to pro-inflammatory cytokines, and it plays a pivotal role in integrating the cell cycle regulatory machinery with metabolic pathways essential for cell growth and survival [43, 44]. These transcription factors not only drive cellular proliferation but are also reported to enhance the immunomodulatory capacity of MSCs. The elevated activity of NF-κB in fetal MSCs has been associated with an enhanced paracrine response. For instance, preconditioning MSCs with H_2_O_2_ resulted in increased NF-κB-dependent expression of growth factors that contribute to their paracrine effects [45]. Moreover, the immunomodulatory potential of MSCs is further amplified in the presence of inflammatory cytokines IFN-γ and TNF-α. Hemeda et al. demonstrated that while these cytokines do not impair the ability of MSCs to suppress T-cell proliferation, IFN-γ specifically upregulates IDO expression, which is critical for immunosuppression [46]. Further insights into cytokine-mediated modulation of MSCs were provided by Lopez et al., who showed that optimal concentrations and timing of TNF-α and IFN-γ enhance the expression of immunoregulatory molecules, leading to the production of extracellular vesicles enriched with these factors [47]. Additionally, the immunosuppressive effects of MSCs can be enhanced through engineering approaches, as demonstrated by Shou et al., who found that IFNα-secreting MSCs exhibit greater tumor-inhibitory effects compared to IFNα alone. The essential role of NF-κB in the functional capabilities of MSCs is highlighted by evidence showing that inhibition of NF-κB signaling completely abolishes the ability of MSC-derived exosomes to induce tubule formation *in vitro* [48]. Additionally, mTORC1 signaling was highly increased in fetal MSCs compared to adult MSCs in our study. Since mTORC1 regulates cellular metabolism and has been linked to immune modulation, the elevated mTORC1 activity in fetal MSCs may facilitate their enhanced metabolic flexibility, which is essential for immune response and tissue repair [44]. In line with the findings in other studies, mTORC1 activation in MSCs could promote glycolytic activity, thus improving their capacity to suppress T-cell proliferation and enhance their immunomodulatory function, particularly in inflammatory environments [49]. These findings suggest that the combined influence of NF-κB and mTORC1 signaling pathways may contribute to the superior immunomodulatory and regenerative potential of fetal MSCs compared to adult MSCs. However, further investigations are needed to validate this relationship and to better understand the specific molecular mechanisms driving the enhanced immunomodulatory and regenerative properties of fetal MSCs.

Fetal MSCs exhibit stronger inhibitory effects on T cell proliferation, differentiation, and activation, as well as enhanced suppression of dendritic cell (DC) maturation and promotion of regulatory T cell (Treg) generation, contributing to a more immune-tolerant state [3, 50–52]. These enhanced immunomodulatory properties are associated with the metabolic profile of fetal MSCs, particularly their regulation of glycolysis. It has been shown that fetal MSCs, including those from the human fetal liver, are more effective in inducing Foxp3 + Tregs and lasting immune modulation than adult bone marrow MSCs [9]. A key feature of fetal MSCs is their metabolic shift, which supports immune-modulatory functions. In the current study, significant upregulation of glycolysis was observed in fetal MSCs, particularly in smumf cells, which also showed higher expression of genes related to cholesterol and sterol biosynthesis, and the interferon and TNF-α signaling pathways. This shift toward glycolysis has been linked to superior immunomodulatory capacity, underscoring the importance of the metabolic profile in the enhanced therapeutic potential of fetal MSCs [54, 55]. Mitochondria play a critical role in MSC functions, including energy metabolism, self-renewal, differentiation, and immune regulation [54]. Glycolysis, crucial for MSC energy production, is especially pronounced during MSC activation, driven by TNF-α and IFN-γ, which enhance glucose consumption and metabolic pathways essential for therapeutic efficacy in inflammatory diseases. Inhibition of glycolysis impairs the expression of immunomodulatory molecules such as IDO and TSG-6, reducing MSC effectiveness [53]. Glycolysis also helps reduce ROS production, enhancing MSC survival in inflammatory conditions and reducing cellular stress. Excess ROS, arising from oxidative phosphorylation, may limit MSC immunomodulatory capacity under oxidative stress [54]. In aged MSCs, reduced expression of key glycolytic enzymes, such as hexokinase 1 (HK1) and phosphofructokinase (PFKM), is observed [56, 57]. Herein, we propose that enhancing glycolysis in fetal MSCs, particularly smumf cells, may be linked to improved energy production, immune modulation, and therapeutic efficacy through the activation of key metabolic and signaling pathways.

In conclusion, this study provides a comprehensive and comparative transcriptomic analysis of MSCs derived from various tissues of origin, including BM, AD, and UC, as well as UC MSCs across early and late passages and isolated using enzymatic or Minimal Cube Explant (MCE; smumf cells) methods. Our findings underscore the distinct RNA profile characteristics of fetal MSCs compared to adult MSCs, revealing enhanced transcriptional stability, immunomodulatory capacity, and metabolic flexibility in fetal MSCs, particularly smumf cells. The minimal transcriptomic changes observed in smumf cells across extended passages highlight their suitability for large-scale clinical applications, maintaining their proliferative and functional integrity. Furthermore, the significant upregulation of glycolysis, cholesterol biosynthesis, and key signaling pathways in fetal MSCs suggests their superior potential for immune modulation and tissue repair. While these findings are promising, several limitations should be noted. First, bulk RNA sequencing captures the average gene expression of a cell population, potentially masking cellular heterogeneity and rare subpopulations. Second, regarding terminology, the term "fetal MSCs" was used to reflect the developmental origin and biological characteristics of UC-derived MSCs. However, we acknowledge that, per NIH regulatory definitions, postnatally collected UC tissue is not classified as fetal. This distinction may cause ambiguity and should be understood in a biological rather than regulatory context. Despite these limitations, our findings strengthen the evidence for fetal MSCs, particularly smumf cells, as a more effective and reliable cell source for therapeutic applications, paving the way for further exploration of their molecular mechanisms and clinical application.

## Supplementary Information

Below is the link to the electronic supplementary material.

Supplementary file 1 (DOCX 1717 KB)

## Data Availability

The data underlying this article will be shared on reasonable request to the corresponding author.
