# Supplementary material for: Comparative Transcriptomic Profiling of Mesenchymal Stem Cells from Distinct Tissue Origins and Isolation Methods Highlights the Stability and Immunomodulatory Signature of Umbilical Cord-Derived Smumf Cells
Source: Tissue Eng Regen Med. 2025 Nov 24;23(1):157–73. doi: 10.1007/s13770-025-00765-2 (PMC12775242; doi:10.1007/s13770-025-00765-2)
Supplement: Supplementary file 1 — (DOCX 1717 KB) [file 13770_2025_765_MOESM1_ESM.docx]

**Supplementary tables**

| **Top DEGs in AD compared to fetal MSCs** | | | | |
| --- | --- | --- | --- | --- |
| **Upregulated DEGs** | | | | |
| Gene | Description | Log_2_lFCl | P-value | FDR |
| JAK1 | Janus kinase 1 | 2.357 | 9.27E-134 | 2.63E-129 |
| EMILIN2 | elastin microfibril interfacer 2 | 5.720 | 5.28E-101 | 3.75E-97 |
| CRLF1 | cytokine receptor like factor 1 | 10.628 | 7.57E-96 | 4.29E-92 |
| ENPP2 | ectonucleotide pyrophosphatase/phosphodiesterase 2 | 8.326 | 3.53E-92 | 1.67E-88 |
| ABHD5 | abhydrolase domain containing 5, lysophosphatidic acid acyltransferase | 2.614 | 3.93E-88 | 1.39E-84 |
| A2M | alpha-2-macroglobulin | 12.304 | 7.17E-88 | 2.26E-84 |
| PCDH18 | Protocadherin 18 | 3.884 | 9.03E-79 | 1.97E-75 |
| CYGB | Cytoglobin | 7.425 | 1.34E-72 | 2.24E-69 |
| TBX15 | T-box transcription factor 15 | 12.859 | 4.84E-72 | 7.64E-69 |
| APBB1IP | amyloid beta precursor protein binding family B member 1 interacting protein | 11.288 | 1.62E-68 | 2.30E-65 |
| **Downregulated DEGs** | | | | |
| Gene | Description | Log_2_lFCl | P-value | FDR |
| MYRF | myelin regulatory factor | 7.010 | 2.35E-126 | 3.34E-122 |
| BDKRB1 | bradykinin receptor B1 | 6.197 | 4.66E-121 | 4.41E-117 |
| PITX2 | paired like homeodomain 2 | 8.148 | 3.87E-88 | 1.39E-84 |
| PLEKHA2 | pleckstrin homology domain containing A2 | 3.313 | 2.82E-86 | 8.01E-83 |
| KRT8 | keratin 8 | 5.839 | 2.47E-84 | 6.36E-81 |
| AMIGO2 | adhesion molecule with Ig like domain 2 | 5.812 | 1.75E-82 | 4.14E-79 |
| KIAA1549 | KIAA1549 | 4.210 | 4.95E-78 | 1.00E-74 |
| PCDH10 | protocadherin 10 | 9.259 | 9.28E-77 | 1.68E-73 |
| IGF2BP1 | insulin like growth factor 2 mRNA binding protein 1 | 4.818 | 9.49E-77 | 1.68E-73 |
| TOR4A | torsin family 4 member A | 6.426 | 2.56E-70 | 3.82E-67 |
| **Top DEGs in BM compared to fetal MSCs** | | | | |
| **Upregulated DEGs** | | | | |
| Gene | Description | Log_2_lFCl | P-value | FDR |
| ITGA7 | interleukin 13 receptor subunit alpha 2 | 7.736 | 1.69E-140 | 4.79E-136 |
| ENPP2 | secreted frizzled related protein 4 | 7.099 | 9.76E-89 | 5.53E-85 |
| KRT16 | peptidase inhibitor 16 | 9.721 | 5.74E-88 | 2.71E-84 |
| LSP1 | protein kinase domain containing, cytoplasmic | 9.262 | 5.59E-80 | 2.26E-76 |
| TBX15 | erythrocyte membrane protein band 4.1 like 3 | 12.908 | 5.80E-73 | 1.83E-69 |
| PLCB4 | prostaglandin-endoperoxide synthase 1 | 4.382 | 1.03E-64 | 2.25E-61 |
| RAB23 | claudin 11 | 1.907 | 2.86E-64 | 5.79E-61 |
| ITGA5 | thrombomodulin | 1.948 | 5.50E-63 | 1.04E-59 |
| ACAN | matrix remodeling associated 5 | 10.564 | 1.12E-58 | 1.86E-55 |
| CRLF1 | matrix metallopeptidase 1 | 8.148 | 1.59E-55 | 2.26E-52 |
| **Downregulated DEGs** | | | | |
| Gene | Description | Log_2_lFCl | P-value | FDR |
| KRT8 | keratin 8 | 8.723 | 3.27E-133 | 4.63E-129 |
| MYRF | myelin regulatory factor | 7.969 | 5.74E-121 | 5.43E-117 |
| BDKRB1 | bradykinin receptor B1 | 6.334 | 5.39E-109 | 3.82E-105 |
| TMEM200A | transmembrane protein 200A | 3.711 | 1.71E-78 | 6.06E-75 |
| COL4A5 | collagen type IV alpha 5 chain | 7.609 | 3.00E-69 | 8.50E-66 |
| DSC3 | desmocollin 3 | 9.040 | 1.29E-66 | 3.33E-63 |
| PITX2 | paired like homeodomain 2 | 4.881 | 8.55E-65 | 2.02E-61 |
| SHC3 | SHC adaptor protein 3 | 6.055 | 4.83E-61 | 8.56E-58 |
| PCDH7 | protocadherin 7 | 6.217 | 1.63E-56 | 2.57E-53 |
| OGFRL1 | opioid growth factor receptor like 1 | 3.613 | 9.13E-56 | 1.36E-52 |

Supplementary table S1. Top 10 DEGs upregulated or downregulated in AD- and BM-MSCs compared to fetal MSCs, meeting criteria of log_2_|FC| > 0.5 and FDR < 0.05. DEGs, differentially expressed genes; MSCs, mesenchymal stem cells; FC, fold change; FDR, false discovery rate.

| **Top DEGs in AD compared to BM MSCs** | | | | |
| --- | --- | --- | --- | --- |
| **Upregulated DEGs** | | | | |
| Gene | Description | Log_2_lFCl | P-value | FDR |
| IGFBP2 | insulin like growth factor binding protein 2 | 9.119 | 2.37E-111 | 1.97E-107 |
| SRGN | serglycin | 4.838 | 3.47E-64 | 1.09E-60 |
| PLCB4 | phospholipase C beta 4 | 2.861 | 2.82E-56 | 7.83E-53 |
| EDIL3 | EGF like repeats and discoidin domains 3 | 4.774 | 1.35E-55 | 3.37E-52 |
| IGF2 | insulin like growth factor 2 | 6.331 | 7.81E-48 | 1.63E-44 |
| ACTA2 | actin alpha 2, smooth muscle | 4.005 | 6.08E-47 | 1.09E-43 |
| AMIGO2 | adhesion molecule with Ig like domain 2 | 4.450 | 1.96E-45 | 2.88E-42 |
| EPHB2 | EPH receptor B2 | 4.384 | 2.15E-44 | 2.83E-41 |
| DYSF | dysferlin | 5.057 | 3.35E-40 | 3.64E-37 |
| COL8A1 | collagen type VIII alpha 1 chain | 4.454 | 2.56E-37 | 2.56E-34 |
| **Downregulated DEGs** | | | | |
| Gene | Description | Log_2_lFCl | P-value | FDR |
| IL13RA2 | interleukin 13 receptor subunit alpha 2 | 8.674 | 1.64E-123 | 4.09E-119 |
| SFRP4 | secreted frizzled related protein 4 | 6.680 | 2.27E-118 | 2.84E-114 |
| PI16 | peptidase inhibitor 16 | 8.273 | 6.72E-77 | 3.36E-73 |
| PKDCC | protein kinase domain containing, cytoplasmic | 4.995 | 1.31E-68 | 5.44E-65 |
| EPB41L3 | erythrocyte membrane protein band 4.1 like 3 | 9.392 | 4.54E-68 | 1.62E-64 |
| PTGS1 | prostaglandin-endoperoxide synthase 1 | 4.474 | 1.38E-51 | 3.14E-48 |
| CLDN11 | claudin 11 | 7.267 | 3.27E-47 | 6.29E-44 |
| THBD | thrombomodulin | 8.702 | 3.53E-46 | 5.89E-43 |
| MXRA5 | matrix remodeling associated 5 | 4.089 | 6.29E-46 | 9.82E-43 |
| MMP1 | matrix metallopeptidase 1 | 7.217 | 3.83E-45 | 5.32E-42 |

Supplementary table S2. Top 10 DEGs upregulated and downregulated in AD MSCs compared to BM MSCs, meeting criteria of log_2_|FC| > 0.5 and FDR < 0.05. DEGs, differentially expressed genes; MSCs, mesenchymal stem cells; FC, fold change; FDR, false discovery rate.

**Supplementary figure**


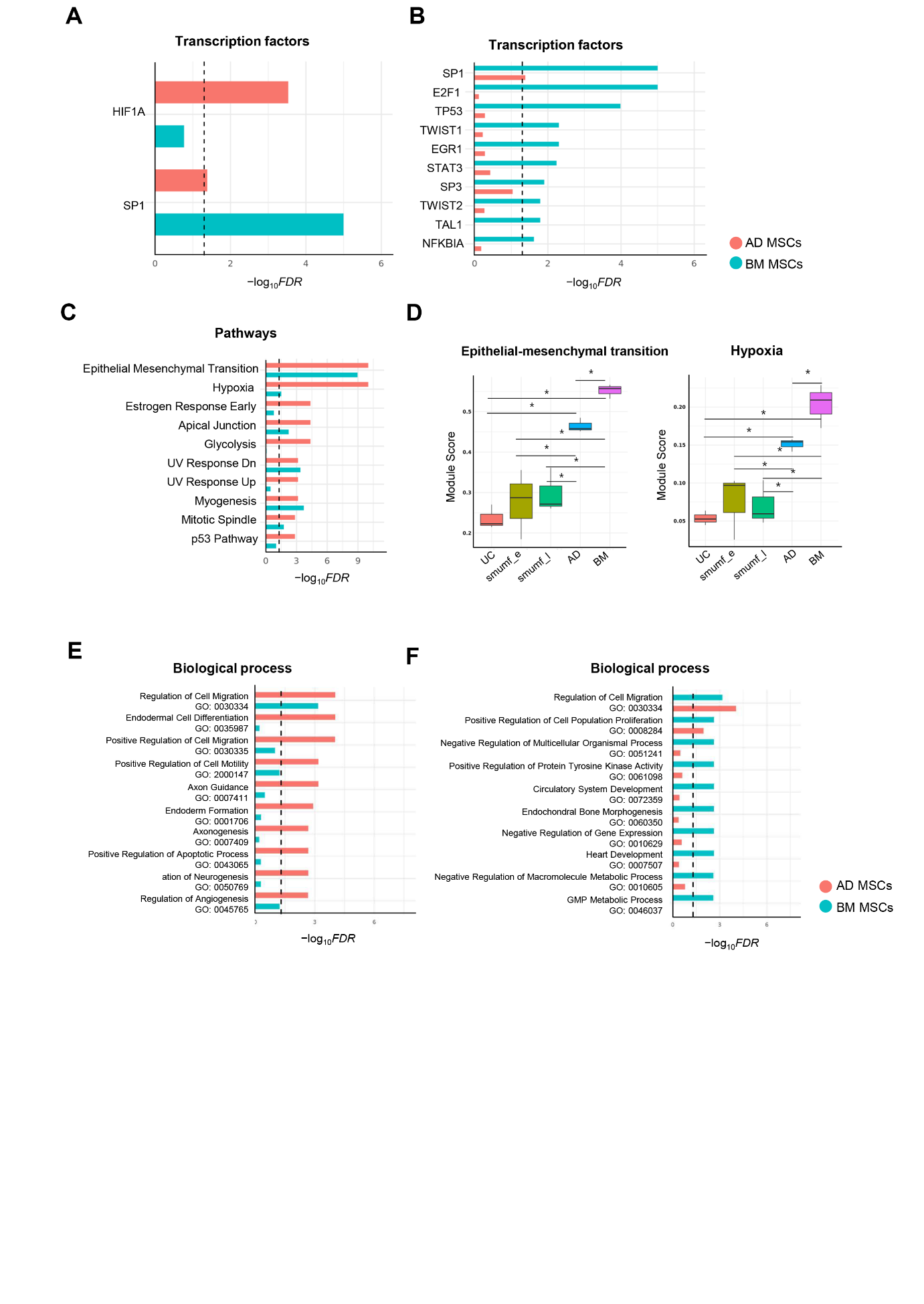


Supplementary figure S1. Comparison between AD and BM MSCs. (A-B) Barplots for transcription factors significantly associated with up- and down-regulated DEGs between AD- and BM-MSCs at FDR 0.05. (C) Barplot representing pathways significantly associated with upregulated DEGs in AD MSCs compared to BM MSCs at FDR 0.05. (D) Boxplots displaying module scores of epithelial-mesenchymal transition (left panel) and hypoxia (right panel) pathways across MSC types. The asterisks indicate that the module scores of the two groups are significantly different at a nominal p-value of 0.05. (E-F) Enriched biological processes for upregulated DEGs in (E) AD- and (F) BM-MSCs. (A-C, E-F) x-axis shows –log_10_(FDR), y-axis displays top up to 10 terms, and the dotted vertical lines represent −log_10_(0.05). Red and turquoise indicate –log_10_(FDR) of corresponding terms for upregulated DEGs in AD- and BM-MSCs, respectively. TFs; transcription factors, FDR; false discovery rate, MSCs; mesenchymal stem cells.


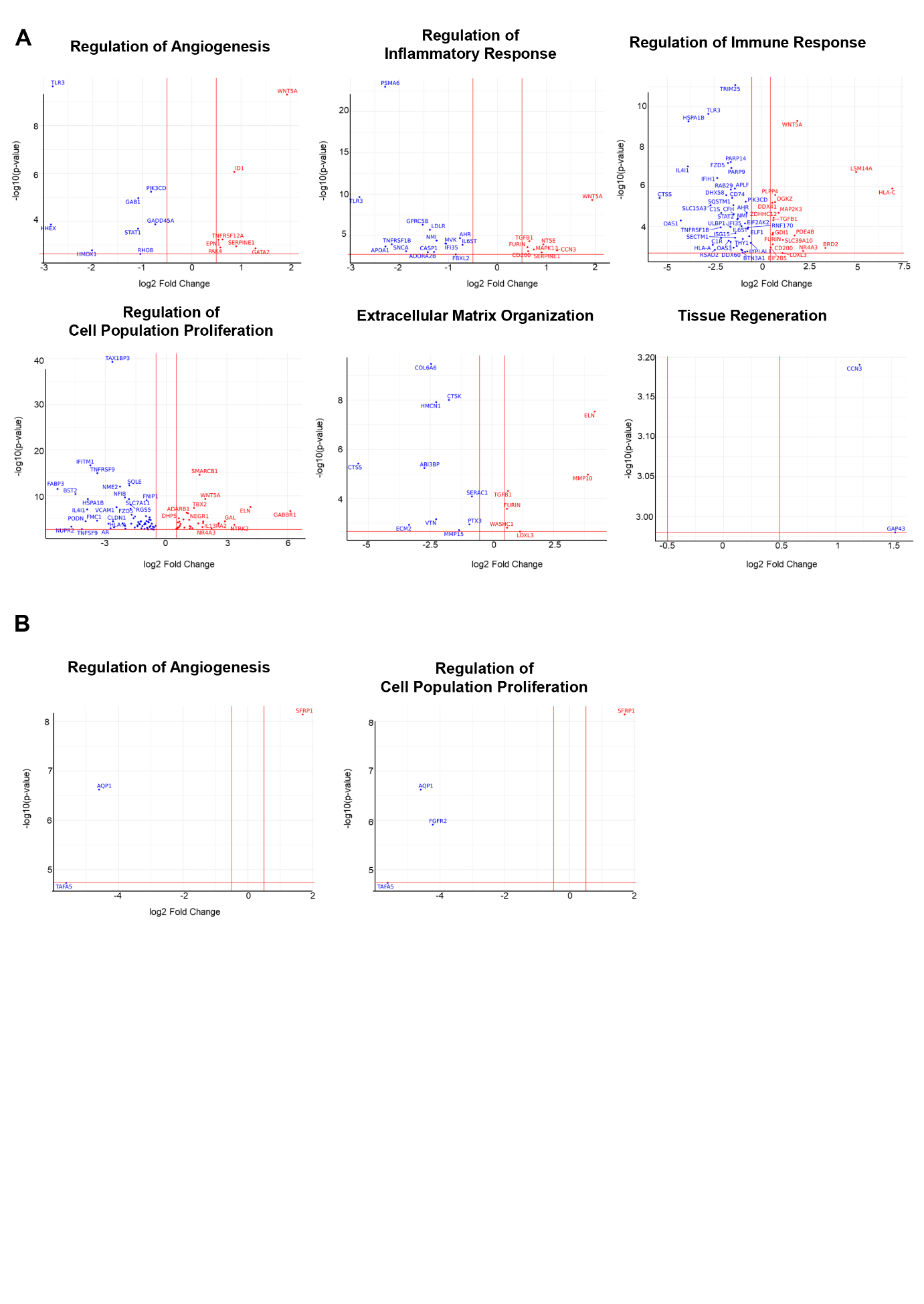


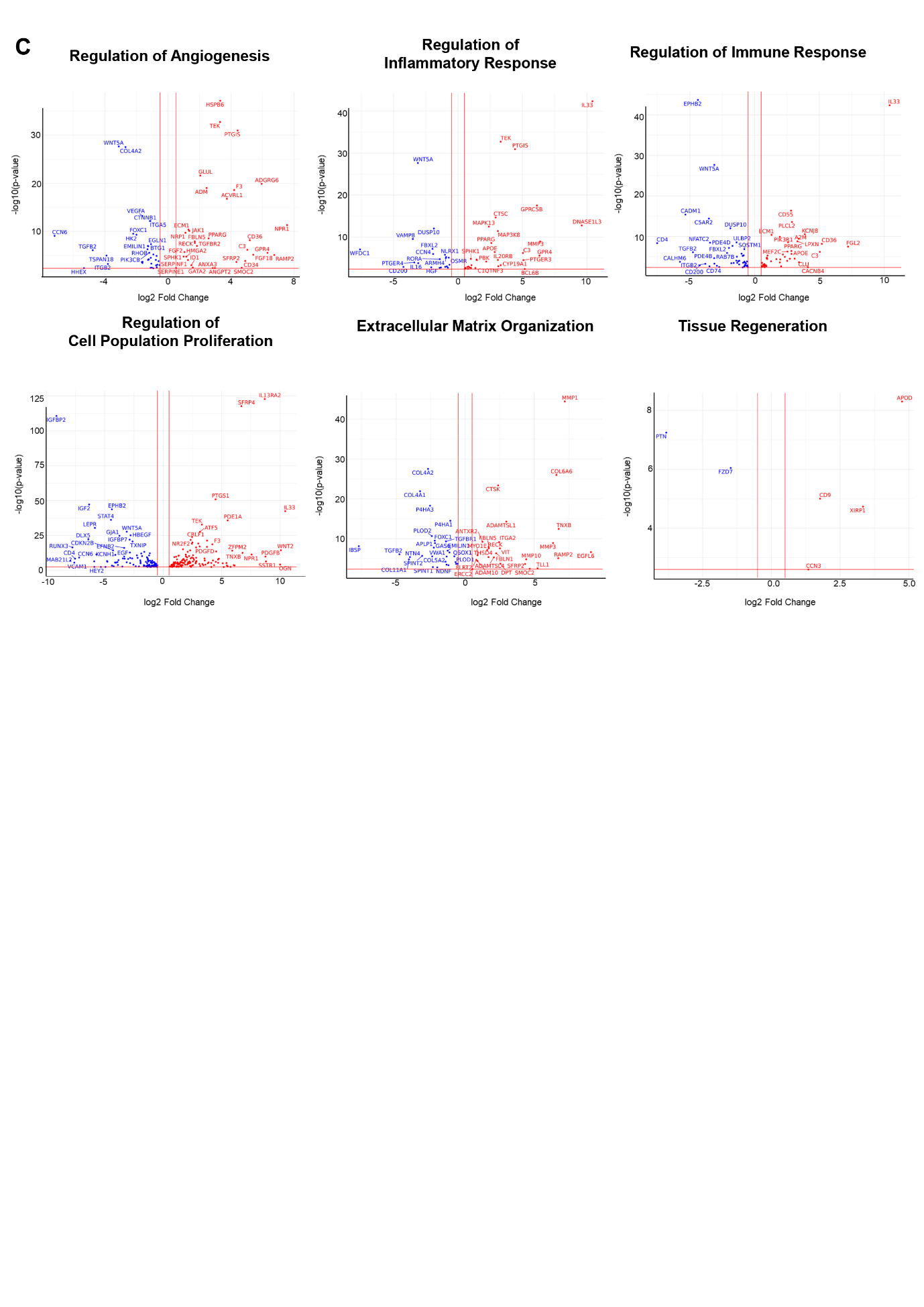


Supplementary figure S2. Volcano plots of DEGs for comparisons within selected biological pathways. (A) smumf and UC mesenchymal stem cells (MSCs), (B) early and late passages of smumf MSCs (only comparisons with DEGs are shown), and (C) BM and AD MSCs. (A-C) The x-axis represents log_2_ fold change, and the y-axis represents -log_10_(p-value). The red horizontal lines show the p-value threshold corresponding approximately to a false discovery rate of 0.05, and the red vertical lines indicate log_2_ fold changes of 0.5 and -0.5. Red and blue dots indicate DEGs significantly up-regulated in (A) UC and smumf MSCs, (B) late and early passages of smumf MSCs, and (C) AD and BM MSCs, respectively.


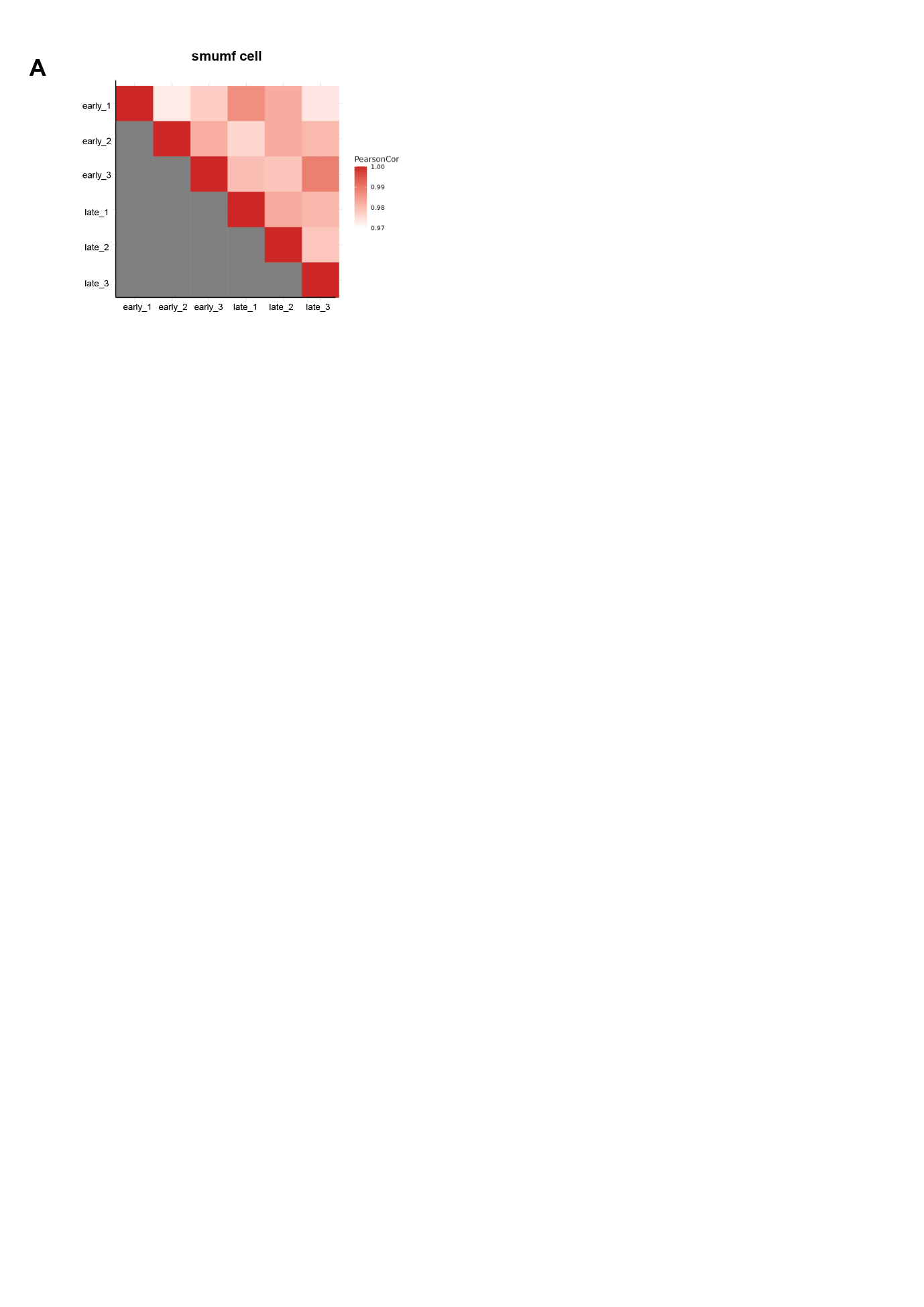


Supplementary figure S3. Tile plot showing Pearson’s correlation coefficients between smumf cells.
